# Supplementary material for: Recursive MAGUS: Scalable and accurate multiple sequence alignment
Source: PLoS Comput Biol. 2021 Oct 6;17(10):e1008950. doi: 10.1371/journal.pcbi.1008950 (PMC8523058; doi:10.1371/journal.pcbi.1008950)
Supplement: S1 Text — Fig A. GCM overview. Fig B. RNASim alignment sizes, MAGUS variants only. Fig C. RNASim alignment error, MAGUS variants only. Fig D. RNASim runtimes, MAGUS guide trees only. Fig E. RNASim runtimes, MAGUS variants only. Fig F. RNASim SPFN error, MAGUS variants only. Fig G. RNASim SPFP error, MAGUS variants only. Fig H. RNASim SPFN error. Fig I. RNASim SPFP error. Fig J. 16S SPFN error. Fig K. 16S SPFP error. Fig L. HomFam (smallest 9 datasets) alignment error. Fig M. Homfam (largest 10 datasets) alignment error. Fig N. Homfam (smallest 9 datasets) runtime. Table A. RNASim log-scale alignment sizes. Table B. RNASim Delta error from lossy compression. Table C. HomFam (all datasets) alignment error. Table D. HomFam (all datasets) SPFN error. Table E. HomFam (all datasets) SPFP error. (PDF) [file pcbi.1008950.s001.pdf]

Recursive MAGUS: scalable and accurate multiple sequence alignment

Vladimir Smirnov[1,*]

1 Department of Computer Science, University of Illinois at Urbana-Champaign, Urbana, IL, United States

* smirnov3@illinois.edu

Supplementary Materials

GCM Overview

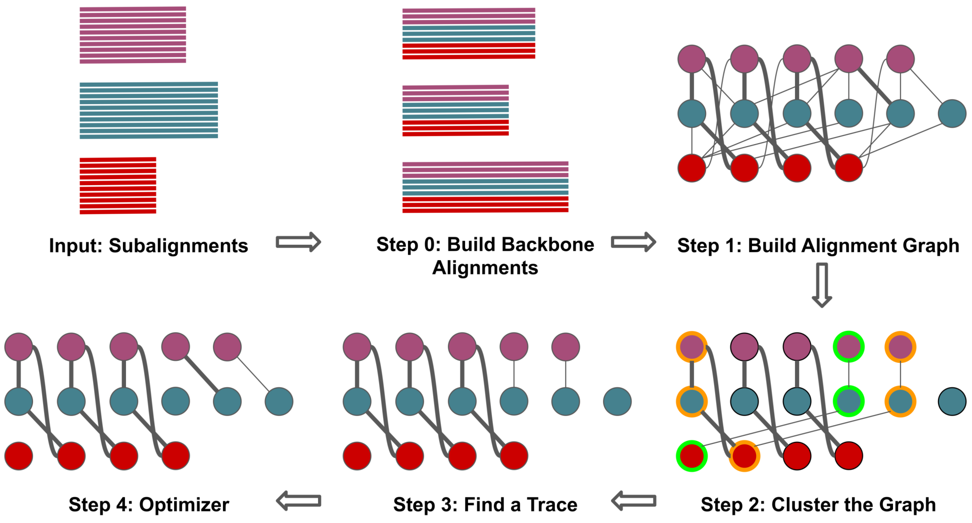

Fig A. GCM overview. Given a set of disjoint subalignments, we first compile (or are given) a set of backbone alignments that span our subsets. These are used to build a weighted alignment graph, where each node is a subalignment column, and the edge weights represent our confidence that these columns should be aligned. Then, we use MCL to cluster the graph. The clusters are further refined into a "trace", which corresponds to a valid multiple sequence alignment. Optionally, the trace can be further optimized with respect to the Maximum Weight Trace criterion.

Preliminary Study: Comparing MAGUS Variants

The Effect of Compression

This part of our study addresses the issues alignment sizes and compression. Fig. B and Table A show the rapid growth of estimated uncompressed MAGUS alignment sizes with increasing dataset size. The size of the true alignment on the full million-sequence

dataset is 21.4 gigabytes, compared to about 3.4 terabytes produced by recursive MAGUS with PartTree and random decompositions, and about 1 terabyte with the other guide trees.

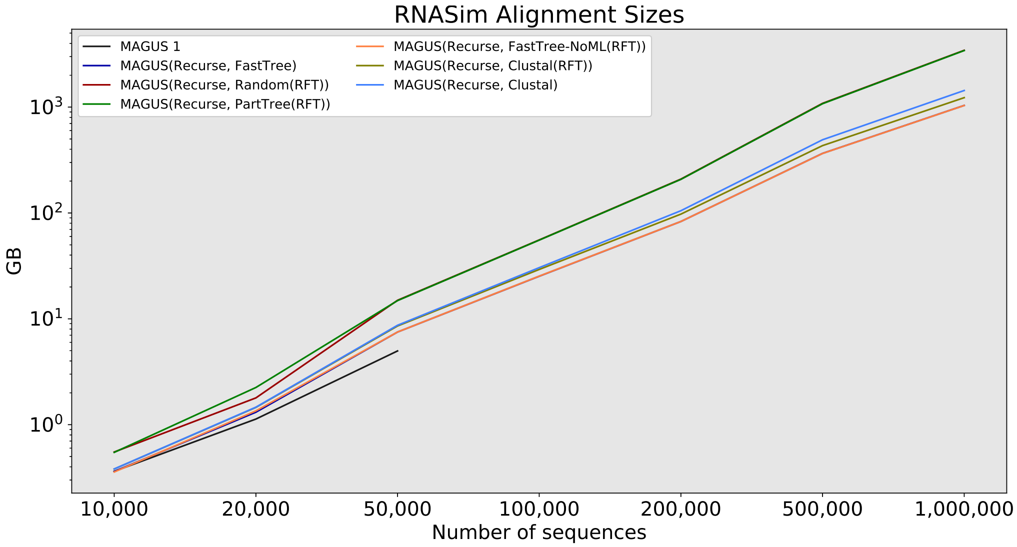

Fig B. RNASim log-scale alignment sizes (in GB), MAGUS variants only. MAGUS was run with 100 subsets on RNASim to reduce load on Blue Waters.

#	M(R,Random)	M(R,PT)	M(R,FT)	M(R,FT-NoML)	M(R,Clustal)	M1
10,000	0.6	0.5	0.4	0.4	0.4	0.4
20,000	1.8	2.2	1.3	1.4	1.5	1.1
50,000	15.0	14.9	7.5	7.5	8.6	5.0
100,000	55.6	55.4	25.3	25.3	29.3	
200,000	208.9	207.6	83.1	83.1	97.5	
500,000	1,085.4	1,076.9	365.5	364.9	432.9	
1,000,000	3,441.8	3,424.4	1,037.3	1,036.5	1,228.5	

Table A. RNASim alignment sizes (in GB) of MAGUS variants. "R" indicates recursion, PT denotes PartTree, FT denotes FastTree. MAGUS was run with 100 subsets on RNASim to reduce load on Blue Waters.

.

Consequently, we measure the increase in SP error from subjecting our alignments to the MAGUS compression. Table B shows the delta SP error of recursive MAGUS (using the FastTree guide tree), with different dataset sizes being compressed down to varying size thresholds. Even under the most aggressive compression policies (e.g. compressing 83 gigabytes to 5), the error increases by minuscule amounts. Looking at the full dataset, we see that compressing the full 1-terabyte alignment to 25 gigabytes only increases the SP error by 1.2e-7 compared to the 100 gigabyte compression. Therefore, we can safely say that our compression scheme for managing huge alignment sizes has negligible effect on alignment accuracy and can be safely used in subsequent experiments.

The Effects of Different Guide Trees

Next, we compare MAGUS 1 to MAGUS with recursion and a selection of different guide trees. Fig C shows the resulting SP error. The runtime analysis is slightly more complicated. We note that the guide tree portion of MAGUS currently always runs on a single compute node, and node parallelism only affects the remaining portion of

	10,000	20,000	50,000	100,000	200,000	500,000	1,000,000
Uncompressed GB	0.4	1.3	7.5	25.3	83.1	365.5	1,037.3
Lossless GB	0.2	0.8	3.7	12.1	40.3	192.6	591.3
100 GB							
50 GB						4.3e-09	3.6e-08
25 GB					1.3e-09	3.6e-08	1.2e-07
10 GB				8.5e-10	1.6e-08	1.8e-07	
5 GB				7.1e-09	1.2e-07		
1 GB			5.9e-08				

Table B. Delta error from lossy compression, MAGUS(Recurse, FastTree) alignments on RNASim. The first two rows indicate the alignment sizes without compression and with only lossless compression, respectively. Subsequent values show the increase in SP error over the uncompressed alignment. 500K and 1M show increase over the 100GB-compressed alignment. Missing values indicate that the alignment could not be compressed to that threshold, or that the lossless alignment was already below it.

MAGUS. Thus, we first show all of the guide tree runtimes separately in Fig D, and the full end-to-end runtime comparison with FastTree and Clustal guide trees in Fig E.

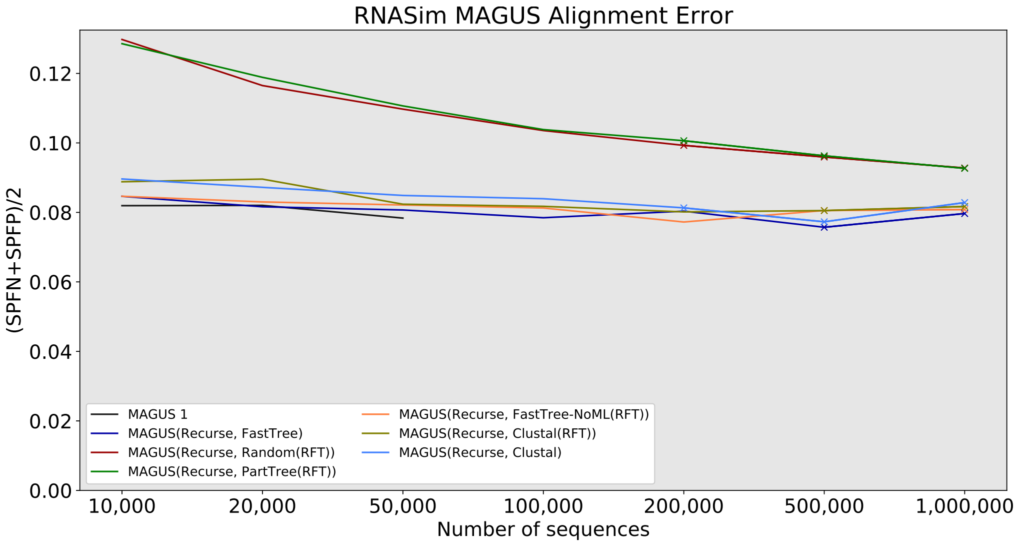

Fig C. RNASim alignment error, MAGUS variants only. Error is the average of SPFP and SPFN. 'X' markers indicate that compression was used (MAGUS alignments above 100GB). 'RFT' denotes that FastTree guide trees were used in recursive subalignments. MAGUS was run with 100 subsets on RNASim to reduce load on Blue Waters.

Guide Tree Runtimes

Firstly, we note that MAGUS 1 wasn't able to proceed past 50,000 sequences, due to out-of-memory issues. MAGUS 1's accuracy is about 0.5% better than MAGUS(Recurse, FastTree) on 10,000 sequences. Notably, where it did finish on 10-50,000 sequences, MAGUS 1 is much faster than MAGUS(Recurse, FastTree) on a single node - about 23 hours vs. about 74 hours.

Comparing recursive MAGUS with different decompositions, we see that the accuracy of using PartTree is about the same as decomposing randomly, with both being predictably the fastest; the PartTree decomposition takes about 4 hours on a

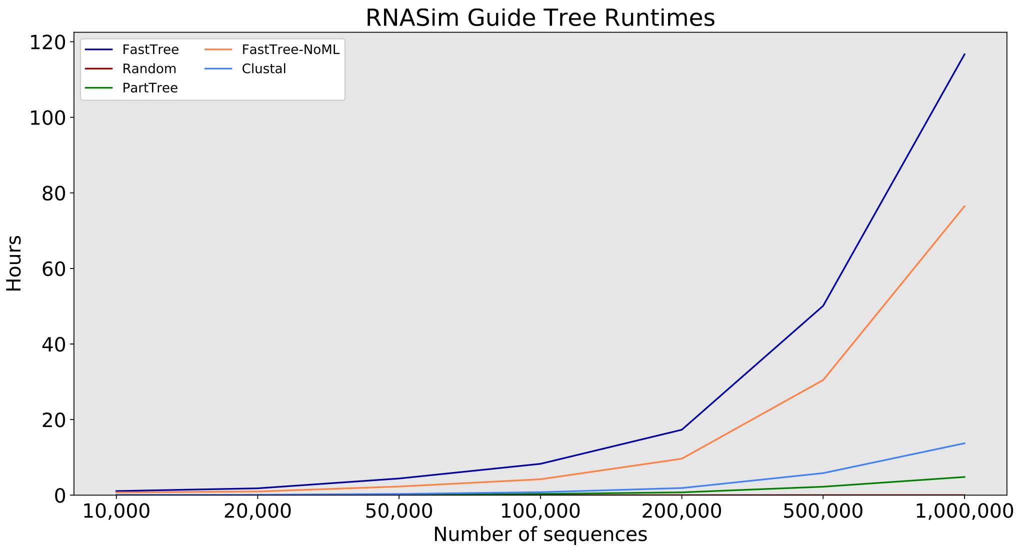

Fig D. RNASim runtimes (in hours) of MAGUS guide trees only.

million sequences, while the random decomposition is nearly instant. Next, we see that using FastTree(no ML) yields about the same accuracy as using Clustal (about 1% better than random), but takes more time on larger datasets: on the full million sequences, it takes about 76 hours to compute the FastTree(no ML) tree, vs. about 14 hours to compute the Clustal Omega tree. Finally, we see that using FastTree seems to give the best accuracy overall, usually giving a small advantage (0-0.5%) over Clustal/FastTree(no ML). Obviously, this is also the most expensive option: the tree takes about 5 days to compute on a million sequences.

From these results, we glean two natural guide tree choices for MAGUS. Using FastTree will constitute our "slowest-but-most-accurate" option, while using Clustal Omega becomes our "fast" option. We choose Clustal Omega, because the resulting accuracy is considerably better than PartTree/random, while the runtime is considerably better than FastTree(no ML), giving us the most practical compromise between speed and accuracy. Consequently, we carry these two variants of MAGUS into our next experiment.

The Impact of Node-Parallelism

Fig E compares the runtime behavior of these two variants, also demonstrating the effects of node parallelism on MAGUS(Recurse, FastTree). On a million sequences, computing a FastTree guide tree takes about 5 days, and the alignment stage on a single node takes about 9 more days. This reduces to about 17 hours on 10 nodes, and about 2.5 hours on 100 nodes. Using Clustal takes about 14 hours for the guide tree, and then about 5 more days to align on a single node.

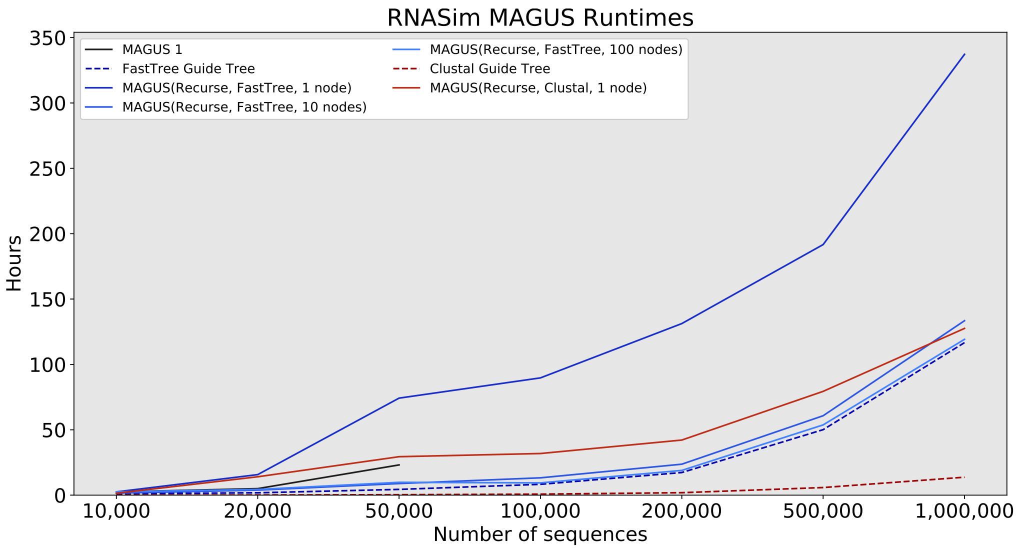

Fig E. RNASim runtimes (in hours), MAGUS variants only. MAGUS was run with 100 subsets on RNASim to reduce load on Blue Waters. Compute nodes were restricted to a maximum walltime of 7 days, but MAGUS(Recurse, FastTree, single node) was run to completion by restarting when time ran out.

Additional Figures

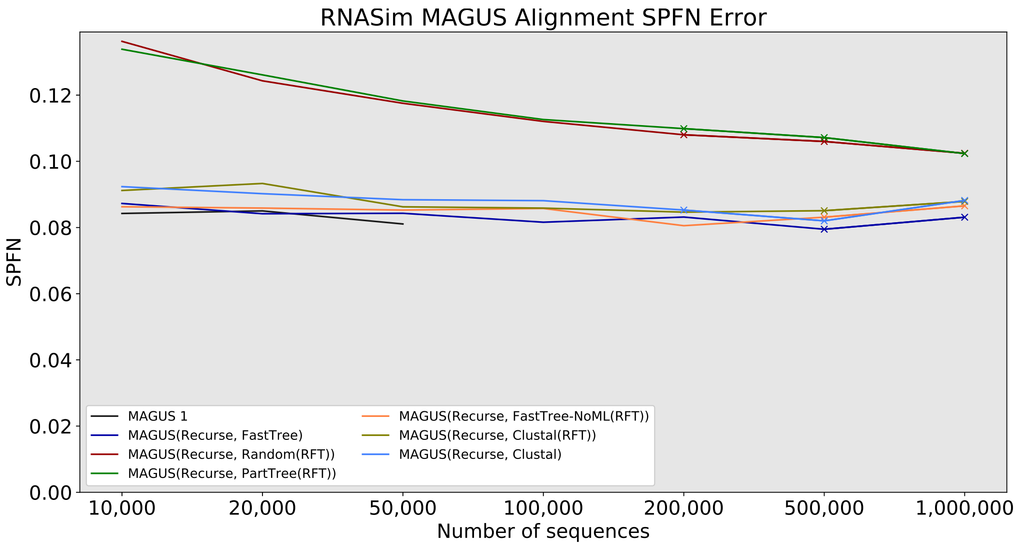

Fig F. RNASim SPFN alignment error, MAGUS variants only. Dashed lines indicate that lossy compression was used (MAGUS alignments above 100GB). MAGUS was run with 100 subsets on RNASim to reduce load on Blue Waters.

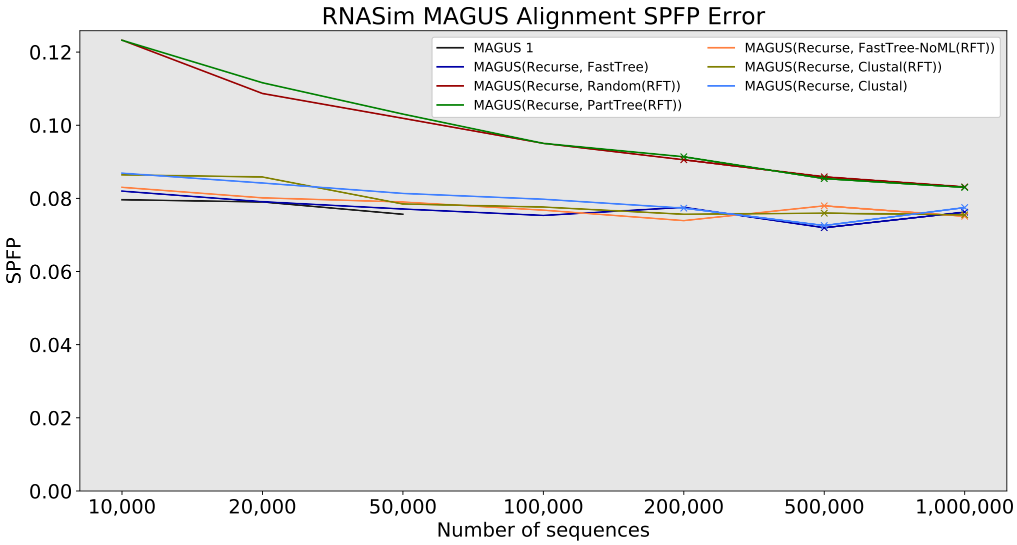

Fig G. RNASim SPFP alignment error, MAGUS variants only. Dashed lines indicate that lossy compression was used (MAGUS alignments above 100GB). MAGUS was run with 100 subsets on RNASim to reduce load on Blue Waters.

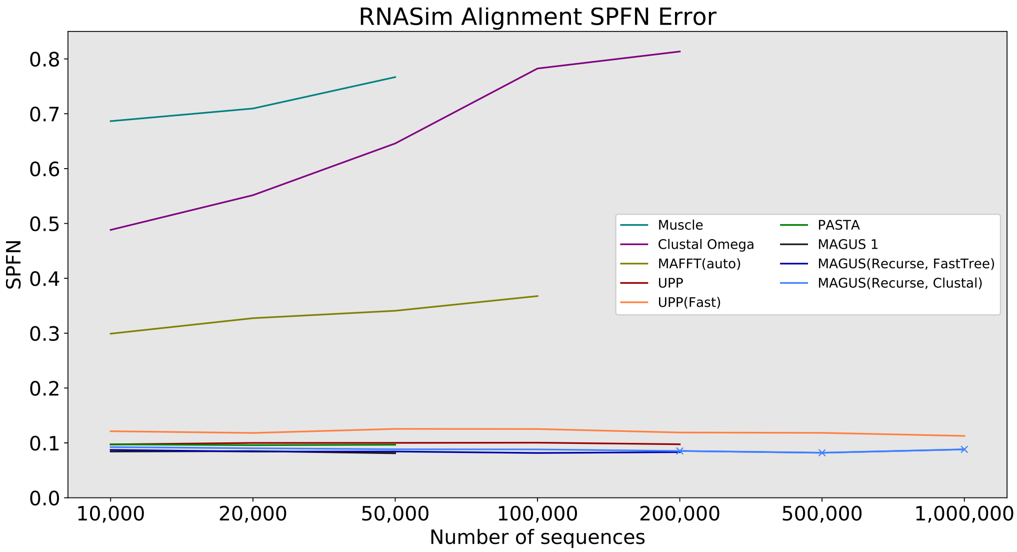

Fig H. RNASim SPFN alignment error, all methods. Dashed lines indicate that lossy compression was used (MAGUS alignments above 100GB). MAGUS was run with 100 subsets on RNASim to reduce load on Blue Waters.

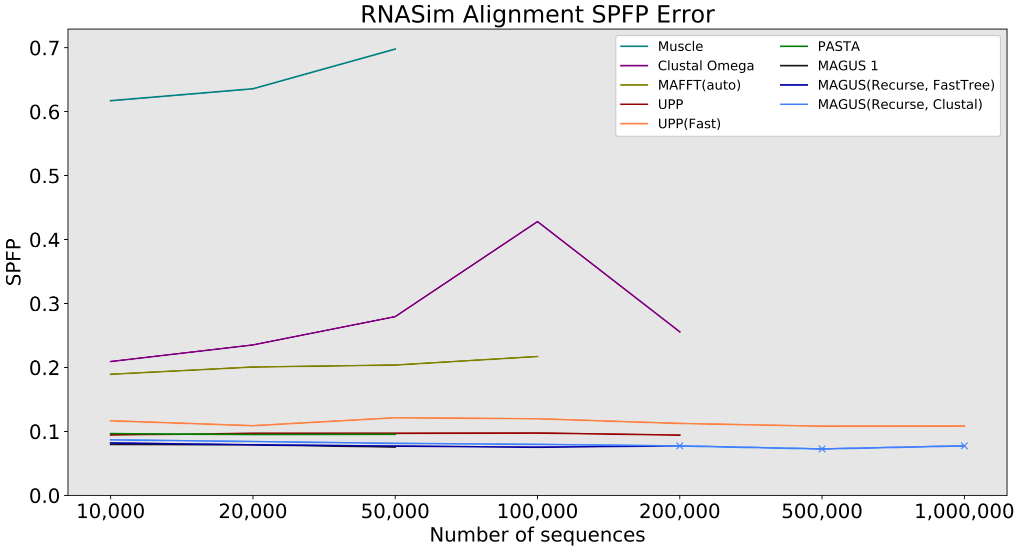

Fig I. RNASim SPFP alignment error, all methods. Dashed lines indicate that lossy compression was used (MAGUS alignments above 100GB). MAGUS was run with 100 subsets on RNASim to reduce load on Blue Waters.

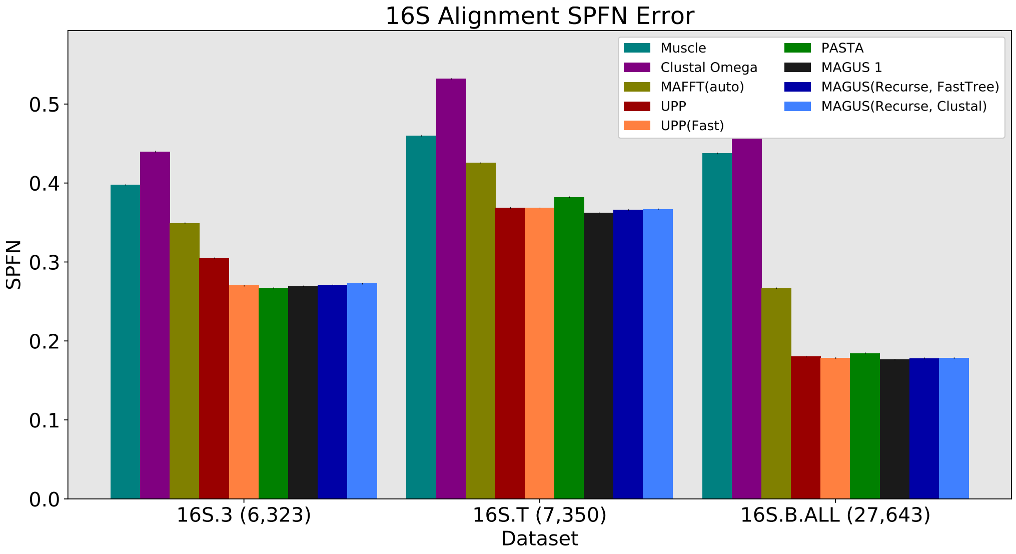

Fig J. 16S SPFN alignment error, all methods. MAGUS was run with the default 25 subsets.

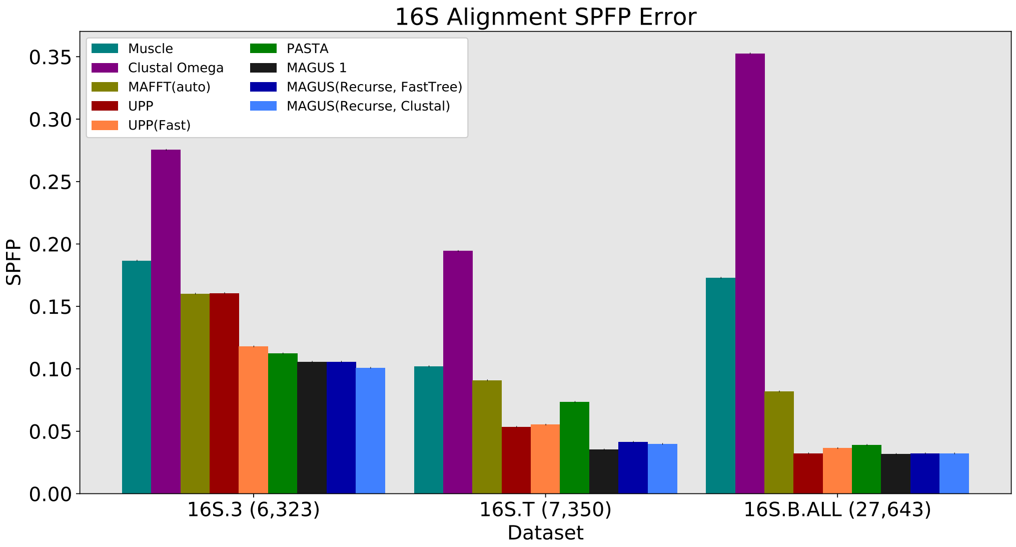

Fig K. 16S SPFP alignment error, all methods. MAGUS was run with the default 25 subsets.

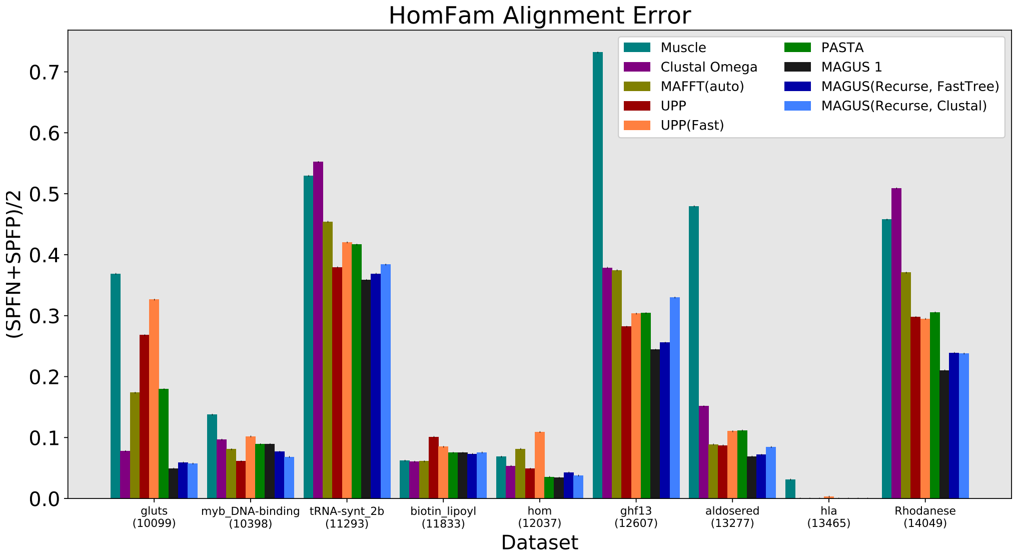

Fig L. HomFam (smallest 9 datasets) alignment error, all methods. Error is the average of SPFP and SPFN. MAGUS was run with the default 25 subsets. (Errors on "hla" are near-zero.)

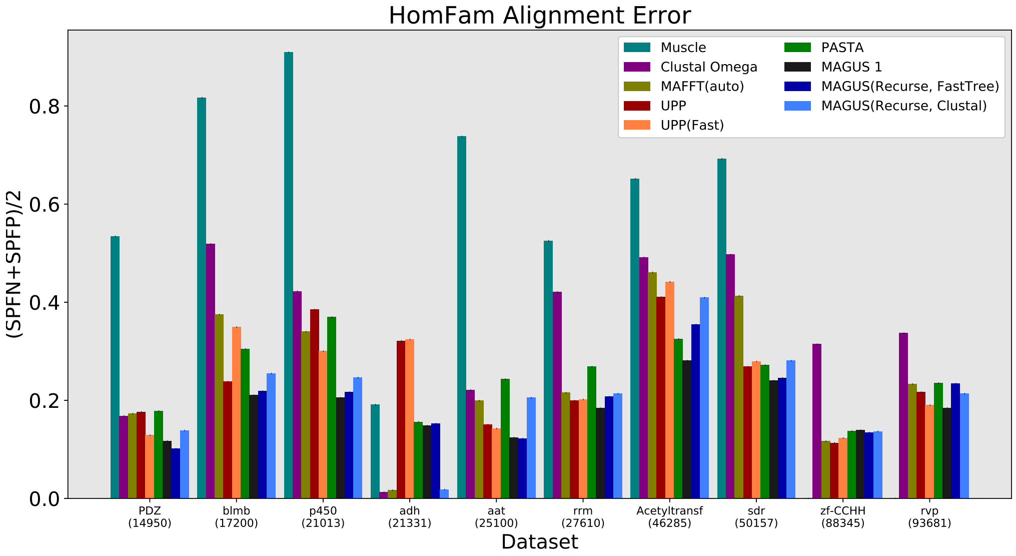

Fig M. Homfam (largest 10 datasets) alignment error, all methods. Error is the average of SPFP and SPFN. MAGUS was run with the default 25 subsets. Muscle segfaulted on the two largest datasets.

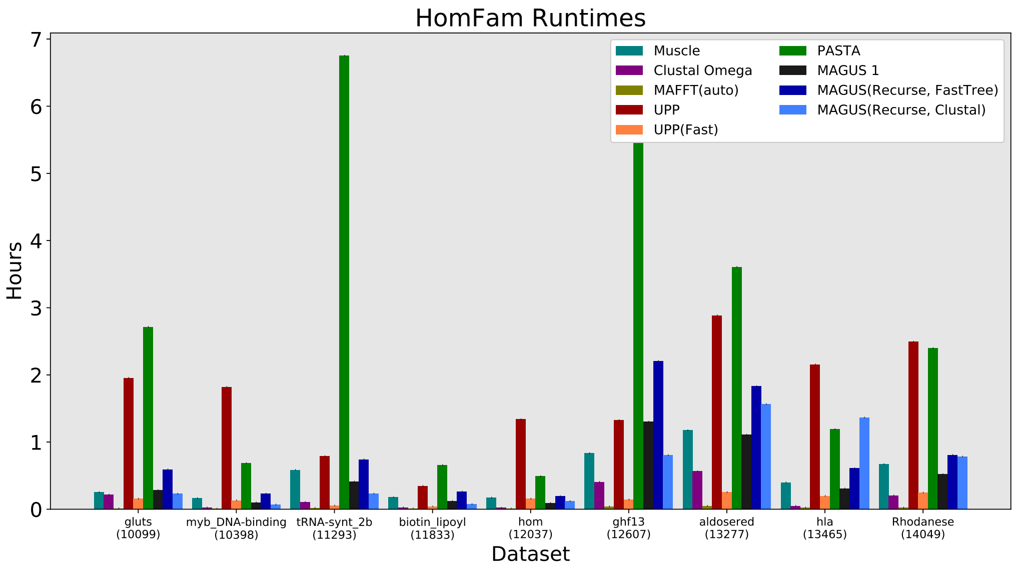

Fig N. Homfam (smallest 9 datasets) runtime, all methods. MAGUS was run with the default 25 subsets.

	Muscle	Clustal	MAFFT	UPP	UPP-F	PASTA	MAGUS 1	MAGUS(R,F)	MAGUS(R,C)
gluts	0.369	0.078	0.174	0.268	0.326	0.179	**0.049**	0.059	0.057
myb-DNA-binding	0.138	0.097	0.081	**0.061**	0.102	0.089	0.089	0.077	0.068
tRNA-synt-2b	0.529	0.552	0.454	0.379	0.420	0.417	**0.359**	0.369	0.384
biotin-lipoyl	0.062	**0.060**	0.061	0.101	0.085	0.075	0.075	0.073	0.076
hom	0.069	0.053	0.081	0.049	0.109	0.035	**0.034**	0.043	0.038
ghf13	0.732	0.379	0.374	0.282	0.303	0.304	**0.244**	0.256	0.330
aldosered	0.480	0.151	0.089	0.087	0.110	0.112	**0.069**	0.072	0.085
hla	0.031	**0.000**	**0.000**	**0.000**	0.003	**0.000**	**0.000**	**0.000**	**0.000**
Rhodanese	0.458	0.509	0.371	0.298	0.295	0.305	**0.210**	0.239	0.238
PDZ	0.534	0.168	0.173	0.176	0.129	0.178	0.117	**0.101**	0.139
blmb	0.817	0.518	0.375	0.238	0.349	0.305	**0.211**	0.219	0.255
p450	0.909	0.422	0.340	0.385	0.300	0.369	**0.205**	0.217	0.246
adh	0.191	**0.013**	0.017	0.321	0.324	0.156	0.148	0.152	0.018
aat	0.738	0.221	0.199	0.150	0.142	0.243	0.124	**0.122**	0.205
rrm	0.525	0.421	0.215	0.199	0.202	0.269	**0.184**	0.207	0.213
Acetyltransf	0.652	0.491	0.461	0.410	0.441	0.325	**0.281**	0.355	0.409
sdr	0.692	0.497	0.413	0.268	0.279	0.271	**0.240**	0.246	0.281
zf-CCHH	segfault	0.314	0.116	**0.113**	0.123	0.137	0.139	0.134	0.137
rvp	segfault	0.337	0.233	0.217	0.190	0.235	**0.184**	0.234	0.214
Average	0.466	0.272	0.228	0.216	0.231	0.214	**0.155**	0.165	0.179

Table C. SP Error over all homfam datasets. MAGUS(R,F) and MAGUS(R,C) indicate recursive MAGUS with FastTree and Clustal, respectively. Error is the average of SPFP and SPFN. MAGUS was run with the default 25 subsets. Values in bold show the best performer on each dataset. The average is shown over the datasets where all methods completed.

	Muscle	Clustal	MAFFT	UPP	UPP-F	PASTA	MAGUS 1	MAGUS(R,F)	MAGUS(R,C)
gluts	0.399	0.093	0.194	0.475	0.524	0.304	**0.048**	0.061	0.059
myb-DNA-binding	0.164	0.140	**0.076**	0.096	0.142	0.118	0.130	0.118	0.106
tRNA-synt-2b	0.522	0.626	0.509	0.543	0.578	0.552	**0.498**	0.511	0.524
biotin-lipoyl	**0.054**	0.064	0.064	0.138	0.124	0.106	0.107	0.115	0.099
hom	0.088	0.070	0.080	0.071	0.193	0.052	**0.050**	0.064	0.054
ghf13	0.785	0.433	0.405	0.382	0.434	0.402	**0.358**	0.384	0.494
aldosered	0.588	0.213	0.091	0.129	0.157	0.146	**0.086**	0.099	0.127
hla	0.042	**0.000**	**0.000**	**0.000**	0.006	**0.000**	**0.000**	**0.000**	**0.000**
Rhodanese	0.544	0.607	0.393	0.386	0.404	0.407	**0.298**	0.350	0.360
PDZ	0.610	0.196	0.184	0.220	0.189	0.207	0.169	**0.157**	0.181
blmb	0.876	0.637	0.411	0.319	0.441	0.357	**0.292**	0.311	0.364
p450	0.947	0.540	0.388	0.619	0.357	0.576	**0.244**	0.274	0.295
adh	0.192	**0.013**	0.017	0.639	0.642	0.221	0.277	0.286	0.024
aat	0.809	0.304	0.218	0.184	0.176	0.291	0.168	**0.167**	0.255
rrm	0.631	0.577	0.227	0.255	0.266	0.325	**0.226**	0.251	0.266
Acetyltransf	0.745	0.634	0.484	0.632	0.662	0.481	**0.462**	0.561	0.621
sdr	0.750	0.623	0.457	0.425	0.438	0.401	**0.366**	0.382	0.429
zf-CCHH	segfault	0.399	**0.143**	0.186	0.192	0.205	0.192	0.197	0.205
rvp	segfault	0.388	0.250	0.242	0.244	0.241	**0.190**	0.254	0.226
Average	0.515	0.339	0.247	0.324	0.337	0.291	**0.222**	0.241	0.251

Table D. SPFN Error over all homfam datasets. MAGUS(R,F) and MAGUS(R,C) indicate recursive MAGUS with FastTree and Clustal, respectively. MAGUS was run with the default 25 subsets. Values in bold show the best performer on each dataset. The average is shown over the datasets where all methods completed.

	Muscle	Clustal	MAFFT	UPP	UPP-F	PASTA	MAGUS 1	MAGUS(R,F)	MAGUS(R,C)
gluts	0.338	0.062	0.153	0.061	0.128	0.055	**0.050**	0.057	0.056
myb-DNA-binding	0.111	0.053	0.085	**0.026**	0.061	0.060	0.048	0.035	0.030
tRNA-synt-2b	0.537	0.478	0.399	**0.215**	0.262	0.281	0.219	0.227	0.243
biotin-lipoyl	0.070	0.057	0.058	0.064	0.046	0.044	0.043	**0.031**	0.052
hom	0.049	0.037	0.082	0.027	0.026	0.019	**0.018**	0.022	0.021
ghf13	0.679	0.325	0.344	0.182	0.172	0.206	0.130	**0.128**	0.165
aldosered	0.372	0.090	0.087	0.045	0.064	0.077	0.052	0.044	**0.042**
hla	0.020	**0.000**	**0.000**	**0.000**	**0.000**	**0.000**	**0.000**	**0.000**	**0.000**
Rhodanese	0.371	0.411	0.348	0.210	0.185	0.204	0.123	0.128	**0.116**
PDZ	0.458	0.140	0.162	0.133	0.069	0.150	0.065	**0.045**	0.096
blmb	0.757	0.400	0.339	0.157	0.257	0.252	0.129	**0.126**	0.145
p450	0.871	0.305	0.292	**0.151**	0.243	0.162	0.167	0.159	0.197
adh	0.191	0.012	0.017	**0.003**	0.006	0.091	0.019	0.018	0.012
aat	0.666	0.138	0.180	0.117	0.109	0.195	0.080	**0.077**	0.155
rrm	0.420	0.264	0.204	0.143	**0.138**	0.212	0.141	0.163	0.160
Acetyltransf	0.558	0.349	0.438	0.188	0.221	0.169	**0.099**	0.148	0.197
sdr	0.634	0.371	0.369	0.112	0.120	0.142	0.114	**0.109**	0.132
zf-CCHH	segfault	0.230	0.090	**0.041**	0.054	0.069	0.086	0.072	0.068
rvp	segfault	0.286	0.216	0.192	**0.136**	0.229	0.178	0.214	0.201
Average	0.418	0.205	0.209	0.108	0.124	0.136	**0.088**	0.089	0.107

Table E. SPFP Error over all homfam datasets. MAGUS(R,F) and MAGUS(R,C) indicate recursive MAGUS with FastTree and Clustal, respectively. MAGUS was run with the default 25 subsets. Values in bold show the best performer on each dataset. The average is shown over the datasets where all methods completed.
