# Supplementary material for: Recursive MAGUS: Scalable and accurate multiple sequence alignment
Source: PLoS Comput Biol. 2021 Oct 6;17(10):e1008950. doi: 10.1371/journal.pcbi.1008950 (PMC8523058; doi:10.1371/journal.pcbi.1008950)
Supplement: Attachment — Submitted filename: review-response.pdf [file pcbi.1008950.s002.pdf]

Dear Dr. Schneidman-Duhovny,

Thank you very much for the time and consideration you've given to reviewing the manuscript. Please find my responses to reviewer comments below, marked in blue. The revised paper also indicates the changes in blue text, for your convenience.

The new additions mainly concern the MAGUS compression scheme, brought up by both reviewers; I added some discussion to the main paper pertaining to compression performance, new functionality to permit lossless compression, some data pertaining to lossless compression, and a new figure demonstrating the compression scheme. I also added some discussion about using large alignments for tree estimation, as requested by the second reviewer. Please let me know if there are any additional changes anyone would like to see.

Thank you again for your time and assistance.

With warmest regards,
Vladimir Smirnov

Reviewer #1:
The author presents an update to a recently-published tool (MAGUS; Smirnov & Warnow, 2020). For context, MAGUS is a divide-and-conquer approach for Multiple Sequence Alignment (MSA) that is similar in spirit to PASTA (Mirarab et al., 2015) in that a complete sequence dataset is decomposed into smaller subsets, and the subsets are aligned and merged into a single MSA using a guide tree. The novelty of MAGUS (from the original manuscript) is the use of a novel "Graph Clustering Merger" approach for merging the subset alignments.

In this manuscript specifically, the author expands upon MAGUS to improve scalability in 4 key ways:

1. Recursion: To better handle ultra-large datasets in which even the subsets are prohibitively large for the underlying aligner (e.g. MAFFT), MAGUS can now recursively call itself on the subsets (i.e., to break them down into *even smaller* subsets). This is a clever idea, and I'm excited to see that it yielded significant speed-up with respect to the original MAGUS approach, but the notion of breaking down a divide-and-conquer algorithm into smaller versions of itself recursively is fairly standard (I would even argue that *all* divide-and-conquer algorithms are inherently recursive, and in this context, the only theoretical change is that the MAGUS "base case" of using MAFFT is being made smaller).

2. Parallelism: Prior to this manuscript, MAGUS supported thread-parallelism (i.e., it could utilize all available threads on a single node), and in this update, MAGUS now also supports node-parallelism (i.e., it can now distribute tasks to multiple compute nodes, with each supporting thread-parallelism). This is an excellent additional feature that I'm excited to see

implemented, but the notion of sending the individual components of a divide-and-conquer algorithm to multiple compute nodes is also fairly standard from a technical standpoint.

3. Guide Tree: In the original manuscript, MAGUS used FastTree to estimate a rough guide tree (via the maximum-likelihood approach) with which to decompose the dataset. In this update, MAGUS now supports more types of trees (e.g. to enable using a faster tree-construction approach at the expense of accuracy). This is a nice addition as far as the software itself is concerned, but from a theoretical standpoint, this is a fairly trivial update (the fundamental approach is still the same, just with the newly-added ability to swap in different tools to construct this initial guide tree).

4. Memory Management and Alignment Compression: To reduce the memory complexity of MAGUS, the author has implemented optimizations on two fronts. First, memory management is conducted more optimally by only fully loading a single subalignment into memory at any given time. This is a nice fix that I'm very excited to see implemented into MAGUS, but this is certainly more of a code revision rather than a novel approach: only loading pieces of a dataset into memory at any given time has been a standard systems programming approach for decades. Second, to *further* improve the memory complexity of MAGUS, the author has now also implemented a lossy compression scheme to "dissolve" neighboring columns that are highly-similar. I actually found the idea quite interesting, and more exploration of how this lossy compression scheme impacts accuracy would have been nice to see, especially as a function of the threshold.

The results are nice to see, but they are unsurprising: nice (but not inherently novel) optimizations were made to the MAGUS codebase, and the MAGUS runtime improved considerably as a result, but with negligible impact to accuracy. From my perspective, while these improvements are excellent from a codebase improvement perspective and are surely welcome to the MAGUS userbase, they seem to be fairly standard approaches and, in the context of the original MAGUS manuscript (which I have been reading side-by-side next to this manuscript), do not seem sufficiently novel beyond the previous MAGUS manuscript to justify publication in PLOS Computational Biology (rather, this article seems more appropriate for a technical blog post or similar).

Thank you very much for your review. I certainly agree that, taken individually, these four scalability improvements are not significant innovations in computer science. You are absolutely correct that these changes would not offer much if judged solely on the basis of their algorithmic novelty.

Rather, the intended contribution of this work is more to demonstrate the leap in alignment capability that they collectively enable. In this context, these changes are not merely incremental improvements to the MAGUS codebase, but allow it to become the most effective large-scale alignment method of any that I am currently aware of. Thus, the contribution of this manuscript is to demonstrate what is hopefully the current state-of-the-art in solving such problems.

I also agree with your request to expand on the compression part of the paper, so I fleshed it out with some additional information. I also implemented the functionality for MAGUS to perform lossless compression only (i.e. the user can request to terminate the compression step as soon as further compression becomes lossy), and added data showing the thresholds for maximum lossless compression at each of the RNASim dataset sizes. Lastly, I also added a figure walking through a simple example (as requested by the second reviewer).

Reviewer #2:
The manuscript "Recursive MAGUS: scalable and accurate multiple sequence alignment" by Vladimir Smirnov describes an extension of the earlier MAGUS alignment package (which itself is an extension of the even earlier PASTA alignment approach). The fundamental idea behind MAGUS and PASTA is straightforward – 1) sequences to align are broken into groups and each group is aligned; 2) the subalignments are then merged. This basic approach and need for good methods of very large-scale multiple sequence alignments is clear – the PASTA method has been cited almost 250 times in the 6 years since it was published. Thus, the important question for this manuscript is: does Recursive MAGUS represent an important and useful extension of these approaches.

I've gone over the results quite carefully and believe the program is both straightforward and potentially quite useful. There is clear evidence that alignments produced by MAGUS are quite good under all settings tested (Figures 3, 5, 6, and 7). Run-times also appeared to be reasonable in general. The options used to run programs are clear. I do have one big question about the lossy compression (discussed on page 4). I would like to see a figure depicting the algorithm (i.e., a flowchart with examples showing the operations on data columns). That is the one part of the paper where, try as I might, I simply could not understand what is being done by the program.

Another more philosophical area that the author might consider discussing is the potential uses of very large alignments. This issue came to me when I was trying to understand the lossy compression issue. For phylogeny it might be better to estimate several alignments, each of which is relatively large but not so large as to require the lossy compression, and then estimate trees from each and combine the trees using a supertree approach. It is not clear to me whether very large alignments would have benefits for studies of molecular evolution. Again, the alignments could be broken down into subsets and analyzed separately. Note that by "very large" I mean large enough to have to invoke lossy compression – it is clear that large alignments are useful.

Finally, I would like to apologize for a delayed review. Some unexpected stresses on my time came up after I agreed to review. I kept thinking I'd get to the review but then got buried under other obligations. Please accept my sincere apology.

Thank you very much for your review.

I agree that the lossy compression scheme was not presented with enough clarity, and I've added more details and results on the subject, as well as an explanatory figure to the manuscript.

You also raised a very valid point regarding the utility of such large alignments, and whether it might be better to analyze them in smaller chunks. I've added a discussion on this topic to the Future Directions portion of the paper. I think this issue deserves its own, much more detailed investigation in the context of estimating trees over very large datasets, which I'm hoping to explore in the near future.
